# De novo truncating *NOVA2* variants affect alternative splicing and lead to heterogeneous neurodevelopmental phenotypes

**DOI:** 10.1002/humu.24414

**Published:** 2022-06-08

**Authors:** Marcello Scala, Nathalie Drouot, Suzanna C. MacLennan, Marja W. Wessels, Magdalena Krygier, Lisa Pavinato, Aida Telegrafi, Stella A. de Man, Marjon van Slegtenhorst, Michele Iacomino, Francesca Madia, Paolo Scudieri, Paolo Uva, Thea Giacomini, Giulia Nobile, Maria Margherita Mancardi, Ganna Balagura, Giovanni Battista Galloni, Alberto Verrotti, Muhammad Umair, Amjad Khan, Jan Liebelt, Miriam Schmidts, Thorsten Langer, Alfredo Brusco, Beata S. Lipska‐Ziętkiewicz, Jasper J. Saris, Nicolas Charlet‐Berguerand, Federico Zara, Pasquale Striano, Amélie Piton

**Affiliations:** ^1^ Department of Neurosciences, Rehabilitation, Ophthalmology, Genetics, Maternal and Child Health University of Genoa Genoa Italy; ^2^ Pediatric Neurology and Muscular Diseases Unit IRCCS Istituto Giannina Gaslini Genoa Italy; ^3^ Institut de Génétique et de Biologie Moléculaire et Cellulaire Illkirch France; ^4^ Centre National de la Recherche Scientifique, UMR7104 Illkirch France; ^5^ Institut National de la Santé et de la Recherche Médicale, U964 Illkirch France; ^6^ Université de Strasbourg Illkirch France; ^7^ Department of Paediatric Neurology Women's and Children's Hospital Adelaide South Australia Australia; ^8^ Department of Clinical Genetics Erasmus Medical Centre Rotterdam The Netherlands; ^9^ Department of Developmental Neurology Medical University of Gdańsk Gdańsk Poland; ^10^ Department of Medical Sciences University of Turin Turin Italy; ^11^ Center for Molecular Medicine Cologne, Institute of Human Genetics University of Cologne Cologne Germany; ^12^ Clinical Genomics Program, GeneDx Gaithersburg Maryland USA; ^13^ Department of Pediatrics Amphia Hospital Breda The Netherlands; ^14^ Unit of Medical Genetics IRCCS Giannina Gaslini Institute Genoa Italy; ^15^ Clinical Bioinformatics Unit IRCCS Istituto Giannina Gaslini Genoa Italy; ^16^ Unit of Child Neuropsychiatry IRCCS Istituto Giannina Gaslini Genoa Italy; ^17^ Struttura Complessa Neuropsichiatria Infantile Sud Azienda Sanitaria Locale Città di Torino Torino Italy; ^18^ Department of Pediatrics University of Perugia Perugia Italy; ^19^ Medical Genomics Research Department, King Abdullah International Medical Research Center (KAIMRC), King Saud Bin Abdulaziz University for Health Sciences Ministry of National Guard Health Affairs Riyadh Saudi Arabia; ^20^ Department of Life Sciences, School of Science University of Management and Technology (UMT) Lahore Pakistan; ^21^ Faculty of Biological Science, Department of Zoology University of Lakki Marwat Lakki Marwat Pakistan; ^22^ South Australian Clinical Genetics Service Women's and Children's Hospital Adelaide South Australia Australia; ^23^ Department of General Pediatrics and Adolescent Medicine, Medical Center and Faculty of Medicine University of Freiburg Freiburg Germany; ^24^ Department of Neuropediatrics and Muscle Disorders, Center for Pediatrics, Faculty of Medicine University of Freiburg Freiburg Germany; ^25^ Unit of Medical Genetics “Città della Salute e della Scienza” University Hospital Turin Italy; ^26^ Clinical Genetics Unit, Department of Biology and Medical Genetics Medical University of Gdańsk Gdańsk Poland; ^27^ Centre for Rare Diseases Medical University of Gdańsk Gdańsk Poland; ^28^ Laboratory of Genetic Diagnostic Hôpitaux Universitaires de Strasbourg Strasbourg France; ^29^ Institut Universitaire de France, Paris Île‐de‐France France

**Keywords:** alternative splicing, myoclonic seizures, neurodevelopmental disorder, *NOVA2*, psychomotor regression, truncating variants

## Abstract

Alternative splicing (AS) is crucial for cell‐type‐specific gene transcription and plays a critical role in neuronal differentiation and synaptic plasticity. De novo frameshift variants in *NOVA2*, encoding a neuron‐specific key splicing factor, have been recently associated with a new neurodevelopmental disorder (NDD) with hypotonia, neurological features, and brain abnormalities. We investigated eight unrelated individuals by exome sequencing (ES) and identified seven novel pathogenic *NOVA2* variants, including two with a novel localization at the KH1 and KH3 domains. In addition to a severe NDD phenotype, novel clinical features included psychomotor regression, attention deficit‐hyperactivity disorder (ADHD), dyspraxia, and urogenital and endocrinological manifestations. To test the effect of the variants on splicing regulation, we transfected HeLa cells with wildtype and mutant *NOVA2* complementary DNA (cDNA). The novel variants NM_002516.4:c.754_756delCTGinsTT p.(Leu252Phefs*144) and c.1329dup p.(Lys444Glnfs*82) all negatively affected AS events. The distal p.(Lys444Glnfs*82) variant, causing a partial removal of the KH3 domain, had a milder functional effect leading to an intermediate phenotype. Our findings expand the molecular and phenotypic spectrum of *NOVA2*‐related NDD, supporting the pathogenic role of AS disruption by truncating variants and suggesting that this is a heterogeneous condition with variable clinical course.

## Graphical abstract

De novo truncating NOVA2 variants lead to heterogeneous neurodevelopmental phenotypes. We identified novel clinical features (psychomotor regression, behavioral disturbances, dyspraxia, and urogenital and endocrinological manifestations), and showed that NOVA2 variants affect alternative splicing events.

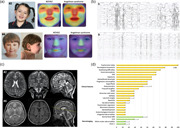

## 1 INTRODUCTION

Proteins involved in the alternative splicing (AS) of genes encoding ubiquitously‐expressed transcriptional regulators have emerged as crucial regulators of cell‐type‐specific transcription, especially in neurons (Lipscombe & Lopez Soto, 2019; Porter et al., 2018; Vuong et al., 2016). Through the inclusion or exclusion of specific alternative exons, AS allows the generation of distinct proteins from a single pre‐messenger RNA (mRNA), contributing to cell‐restricted transcriptional regulation (Lee & Rio, 2015; Park et al., 2018). Complex and unique AS patterns occur in neuronal cells, in which AS is essential for every stage of the life cycle and plays a pivotal role in early differentiation, axonal guidance, synapse formation and plasticity, and even programmed cell death (Lipscombe & Lopez Soto, 2019; Porter et al., 2018; Traunmüller et al., 2016; Vuong et al., 2016; Weyn‐Vanhentenryck et al., 2018). Neuron‐specific AS relies on the coordinate actions of multiple brain‐specific RNA‐binding proteins (RBPs), whose deficient regulation is implicated in the pathogenesis of several neurological disorders (Porter et al., 2018; Vuong et al., 2016; Wilkinson et al., 2020; Will & Lührmann, 2011).

Among neuron‐specific key splicing factors, Neuro‐Oncological Ventral Antigens (NOVAs) dynamically regulate alternative polyadenylation in the brain and the exon content of RNAs encoding crucial proteins for synaptic plasticity (Licatalosi et al., 2008; Ule et al., 2003, 2006, 2005). *NOVA1* (OMIM *602157) and *NOVA2* (OMIM *601991) paralogues encode two highly homologous proteins with three K homology (KH)‐type RNA‐binding domains (KH1‐3), through which they bind the YCAY motifs in the mRNA (Jensen et al., 2000). *NOVA1* is preferentially expressed in the hindbrain and ventral spinal cord, whereas *NOVA2* expression is predominant in the forebrain and dorsal spinal cord (Saito et al., 2016; Vuong et al., 2016; Yang et al., 1998). The pivotal role of NOVAs in the development of peripheral and central nervous system is highlighted by the knock‐out mouse models, showing motor impairment, neuronal apoptosis, long‐term potentiation deficiency, and early lethality (Huang et al., 2005; Jensen et al., 2000; Ruggiu et al., 2009).

NOVA2 is specifically implicated in the splicing regulation of genes involved at different levels in brain development and function (axonal guidance and projection, synaptic formation and plasticity, and Purkinje cells function) (Saito et al., 2019; Vuong et al., 2016). In humans, NOVA2‐related neurodevelopmental disorder (NDD) results from de novo frameshift variants clustered between Ala 241 and Val261 and replacing the KH3 domain by the same alternative C‐terminal part (Mattioli et al., 2020). This condition is characterized by psychomotor delay, cognitive impairment, hypotonia, neurologic features, and brain MRI abnormalities (NDD with or without autistic features and/or structural brain abnormalities—NEDASB, OMIM #618859) (Mattioli et al., 2020). Based on the clustering and type of the reported variants, mutational mechanism was suspected to be either a hypomorphic or gain‐of‐function effect (Mattioli et al., 2020).

Here, we report eight new patients harboring novel truncating variants in *NOVA2*. In addition to four frameshifts clustered in the same protein region previously described (p.(Leu252Phefs*144), p.(Leu252Profs*141), p.(Ala263Profs*133), and p.(Leu276Cysfs*120)), we also identified three novel variants (p.(Gln86*), p.(Leu175Cysfs*6), and p.(Lys444Glnfs*82)) located in KH1, KH2, or KH3 domains. Our data refine the molecular and phenotypic spectrum of *NOVA2* variants and suggest a novel interpretation of disease pathogenicity.

## 2 MATERIALS AND METHODS

### 2.1 Editorial policies and ethical considerations

This study adheres to the principles set out in the Declaration of Helsinki. The following Research Ethics Committees approved the study: Gaslini Children's Hospital (Comitato Etico della Regione Liguria (163/2018) and Città della Salute e della Scienza University Hospital (0060884). No institutional review board (IRB) approval was necessary for retrospective data analysis of a single patient for the following Institutions: Center for Pediatrics and Faculty of Medicine, University of Freiburg, Freiburg (Germany); Erasmus Medical Centre, Rotterdam (The Netherlands); Medical University of Gdańsk, Gdańsk (Poland); University of Lakki Marwat, KPK (Pakistan) University of Management and Technology, Lahore, Punjab (Pakistan); Women's and Children's Hospital, Adelaide (Australia). The authors obtained and archived written informed consents from parents or legal guardians of the enrolled subjects to publish genetic and clinical data, including clinical photographs (#2 and #4) and brain magnetic resonance imaging (MRI) images (#2 and #6).

### 2.2 Subject enrolment and phenotyping

Following the identification through exome sequencing (ES) of a de novo truncating variant in *NOVA2* in a patient with psychomotor delay, behavioral disturbances, and sleep disorders, we collected data from additional individuals harboring de novo *NOVA2* variants through GeneMatcher (Sobreira et al. 2015). Patients were recruited from several clinical and research centres in Australia (Women's & Children's Hospital, South Australia), Europe (Center for Pediatrics and Faculty of Medicine, University of Freiburg, Germany; Erasmus Medical Centre, The Netherlands; Gaslini Children's Hospital, Italy; Medical University of Gdańsk, Poland; University of Turin, Italy), and Middle East (King Abdullah International Medical Research Center, Saudi Arabia). Written informed consent was obtained from the parents or legal guardians of all enrolled subjects. Patient data were anonymized before sharing.

Detailed phenotypic information concerning the developmental history, behavioral disturbances, neurological examinations, and electro‐clinical findings were provided by the referring physicians. Brain magnetic resonance imaging (MRI) scans were locally performed during routine patient care. All articles indexed in PubMed (https://pubmed.ncbi.nlm.nih.gov/?term=itpa) between April 2020, when frameshift *NOVA2* variants were first associated with a human neurodevelopmental condition by Mattioli et al., and July 2021 were retrieved using the terms “*NOVA2*,” “Frameshift variants,” and “Neurodevelopmental disorders.” Previously reported subjects (Mattioli et al., 2020) were critically reviewed in terms of molecular, clinical, and neuroradiological spectrum, and compared with the current cohort.

### 2.3 Variant identification and analysis

ES was performed on genomic DNA extracted from peripheral blood leukocytes using standard local protocols (Supporting Information Material). The identified variants were filtered according to minor allele frequency ≤ 0.001 in the Genome Aggregation Database (gnomAD; https://gnomad.broadinstitute.org) (Lek et al., 2016), presence in ClinVar (https://www.ncbi.nlm.nih.gov/clinvar/), conservation (Genomic Evolutionary Rate Profiling—GERP, http://mendel.stanford.edu/SidowLab/downloads/gerp/) (Cooper et al., 2005), and predicted impact on protein structure and function. In silico tools were employed to predict the pathogenicity of candidate variants using the Ensebl Variant Effect Predictor (VEP) pipeline (https://www.ensembl.org/info/docs/tools/vep/index.html), including Combined Annotation Dependent Depletion (CADD, GRCh37‐v1.6 version, https://cadd.gs.washington.edu), Sorting Intolerant From Tolerant (SIFT, https://sift.bii.a-star.edu.sg), and Polyphen‐2 (http://genetics.bwh.harvard.edu/pph2/) (Adzhubei et al., 2010; Ng, 2003; Rentzsch et al., 2019; Schwarz et al., 2014). Candidate variants were eventually classified according to the American College of Medical Genetics and Genomics and the Association for Molecular Pathology (ACMG/AMP) guidelines (Richards et al., 2015). Sanger sequencing was performed for the validation of the detected variants and the segregation analysis. All *NOVA2* variants are reported according to RefSeq NM_002516.4 (GenBank NC_000019.10), using HGVS recommendations (den Dunnen et al. 2016). All variants were submitted to the Leiden Open Variation Database (LOVD, https://www.lovd.nl) with the following accession numbers: #0000797459, #0000797460, #0000797461, #0000797462, #0000797463, #0000797464, and #0000841619. Further details are available in Supporting Information Material.

### 2.4 In vitro assay to test *NOVA2* variants effect

HeLa cells were transfected as previously described with pcDNA3.1 plasmids containing optimized sequences of human wild‐type and mutant *NOVA2* cDNA tagged with green fluorescent protein (GFP) and a plasmid containing a FLAG‐protein (Mattioli et al., 2020). The mutant sequences include one variant reported by Mattioli et al. (p.Val261Glyfs*135, alias Mut1) or the following variants reported in this study: c.754_756delinsTT (p.Leu252Phefs*144) and c.1329dupC (p.Lys444Glnfs*82). Proteins were extracted and separated on a 10% acrylamide gel, visualized using an in‐house mouse anti‐GFP antibody (1:10,000) and normalized using a FLAG staining (FLAG antibody: 1:1,000; F1804, Sigma‐Aldrich). To test the effect of variants on splicing regulation, mRNA was extracted from transfected HeLa cells and reverse‐transcribed as described (Mattioli et al., 2020). PCR was performed with primers used previously to amplify *APLP2* Exon 12 (24 cycles), *SORBS1* Exon 3 (37 cycles), and *SGCE* Exon 9 (26 cycles), and the PCR products were analyzed by migration on a 2100 Bioanalyzer instrument (Agilent Technologies).

## 3 RESULTS

### 3.1 *NOVA2* variants cluster within or next to the KH domains

**Figure 1:**
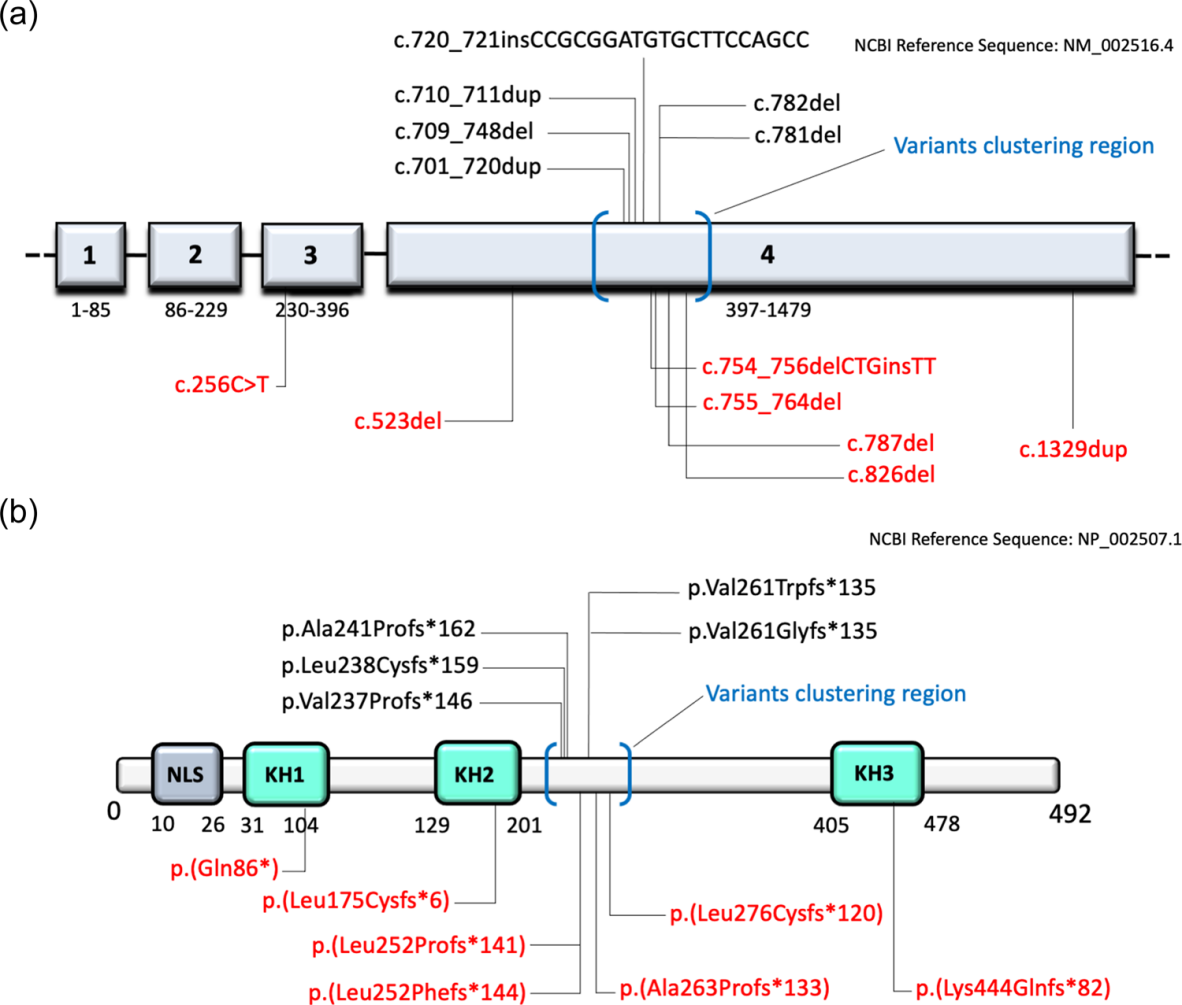
Distribution of *NOVA2* variants. Localization of truncating variants (a) across the exons of *NOVA2* (NCBI Reference Sequence: NM_002516.4; https://www.ncbi.nlm.nih.gov/nuccore/NM_002516.4) and (b) in relation to the K homology (KH)‐type RNA‐binding domains (KH1‐3) domains of NOVA2 protein (NCBI Reference Sequence: NP_002507.1; https://www.ncbi.nlm.nih.gov/protein/NP_002507.1). Previously reported variants are indicated in black above the schematic representation of the gene and the protein, novel variants are reported in red below. The round brackets in blue indicate the potential *NOVA2* “variants clustering region” based on the currently available information, with most of the variants falling in the exons 4 (a) and localizing just after the KH2 domain (b). NLS, nuclear localization signal.

ES led to the identification of seven novel truncating variants in *NOVA2* in our cohort (Figure 1). Three clustered in proximity to those previously reported and add a similar C‐terminal tail: c.787delG p.(Ala263Profs*133) in subject #1, c.754_756delCTGinsTT p.(Leu252Phefs*144) in subject #2, c.826del p.(Leu276Cysfs*120) in subject #4, and c.755_764del p.(Leu252Profs*141) in subjects #7 and #8. We also found three variants located in different protein regions: in subject #3, the distal frameshift c.1329dupC p.(Lys444Glnfs*82) in the KH3 domain; in subject #5, the frameshift variant c.523delC p.(Leu175Cysfs*6) in the KH2 domain; in subject #6, the nonsense variant c.256C>T p.(Gln86*) in the KH1 domain (Figure 1). All these variants are absent in gnomAD database and predicted damaging by in silico tools (Supporting Information: Table 1). No additional potentially causative variants were identified in the studied subjects. All variants are located in the huge last Exon 4 and are predicted to escape nonsense‐mediated mRNA decay (NMD) except c.256C>T p.(Gln86*), located in Exon 3, which is likely to activate NMD leading to haploinsufficiency or to a truncated nonfunctional protein.

### 3.2 *NOVA2* variants affect AS events

**Figure 2:**
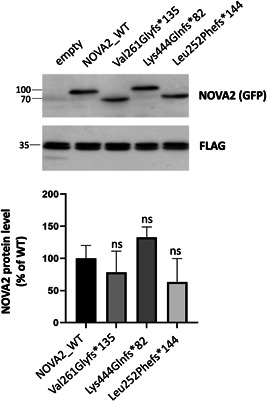
Expression of NOVA2 mutant proteins in HeLa cells. HeLa cells were cotransfected with EGFP‐tagged NOVA2 wild‐type (WT) or mutant cDNA and a plasmid with a FLAG‐tagged protein as a transfection. Proteins were extracted 24 h after transfection, and expression of NOVA2 was analyzed (SDS–PAGE and immunoblotting) using anti‐GFP and anti‐FLAG antibodies. Quantification of NOVA2 protein level was performed from three independent experiments and normalized on FLAG‐protein level. The error bars indicate the standard error mean (SEM). Kruskal–Wallis ANOVA with Dunn's correction for multiple comparisons was performed. ANOVA, analysis of variance; cDNA, complementary DNA; ns, nonsignificant; SDS–PAGE, sodium dodecyl sulfate–polyacrylamide gel electrophoresis.

**Figure 3:**
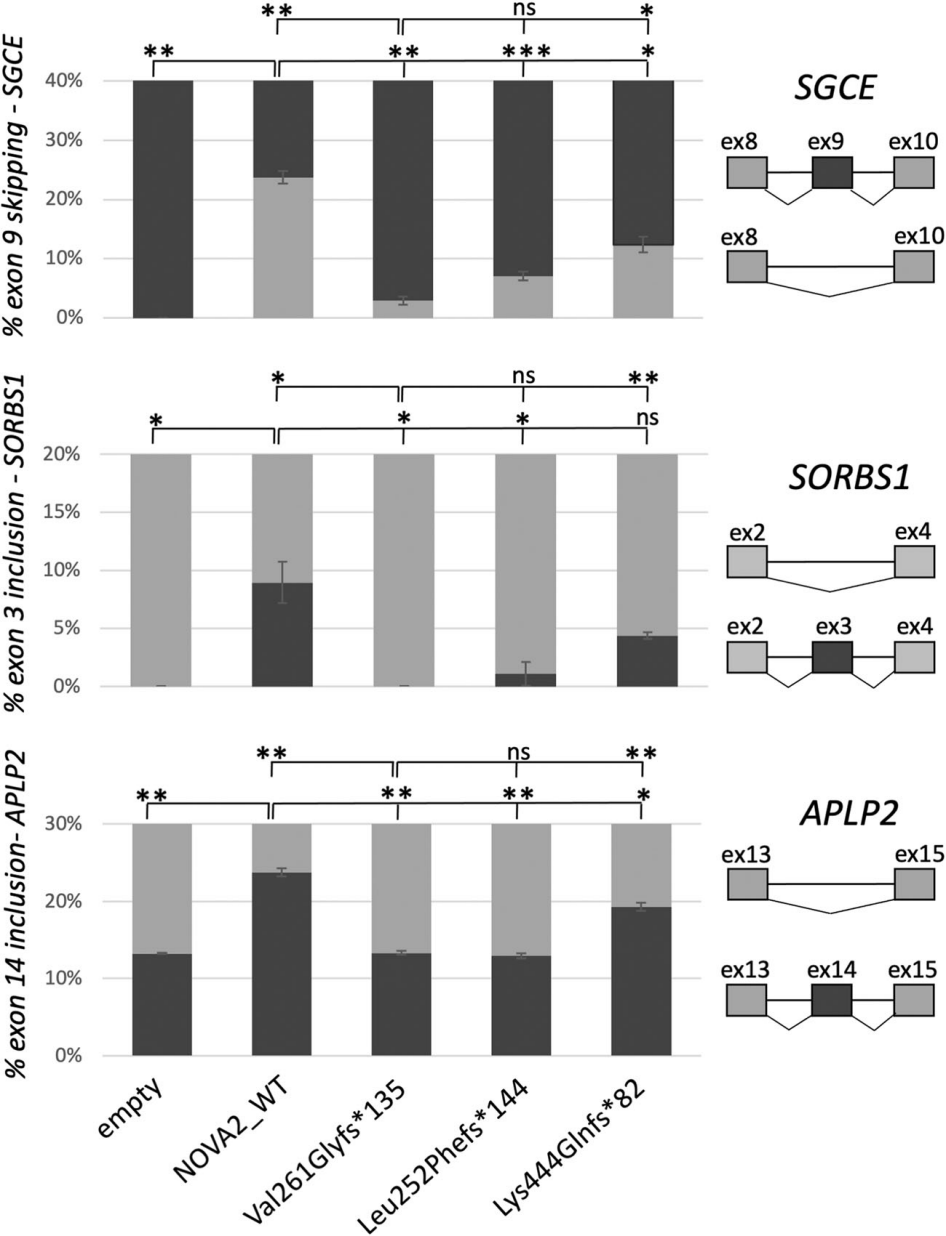
Effect of variants Leu252Phefs*144 and Lys444Glnfs*82 on alternative splicing events regulated by NOVA2. The pcDNA3 eGFP plasmids containing the following NOVA2 cDNA were transfected in HeLa cells: wild‐type (WT), variant reported by Mattioli et al. (Val261Glyfs*135, alias Mut1), c.754_756delinsTT, p.Leu252Phefs*144 variant and c.1329dupC, p.Lys444Glnfs*82 variant. Effects of WT and variant NOVA2 overexpression on alternative splicing events (regulating inclusion of SGCE Exon 9, SORBS1 Exon 3, and APLP12 Exon 14) were analyzed by RT‐PCR and migration on a 2100 Bioanalyzer instrument (Agilent Technology). Three series of experiments were analyzed. The error bars indicate the standard deviation. One‐way ANOVA with Dunnett's multiple comparisons. ANOVA, analysis of variance; ns, nonsignificant; RT‐PCR, reverse‐transcription polymerase chain reaction.***p < .001, *p < .05.

To check the consequences of the identified variants, we introduced them in *NOVA2* complementary DNA (cDNA). We included in the analysis the previously reported c.782del p.(Val261Glyfs*135), the c.754_756delinsTT p.(Leu252Phefs*144) variant, which leads to a similar C‐terminal tail, and the distal frameshift c.1329dupC p.(Lys444Glnfs*82), predicted to remove only part of the KH3 domain. When overexpressed in HeLa cells, no significant difference could be observed in the level of mutant NOVA2 proteins as compared with wild‐type (WT) NOVA2 protein (Figure 2). As previously described, the overexpression of WT *NOVA2* cDNA into HeLa cells leads to significant changes in several AS events: an increase of Exon 9 skipping in *SGCE* transcripts and an increase of Exon 3 and Exon 14 inclusion in *SORBS1* and *APLP2* transcripts, respectively. These changes are not observed when the NOVA2 mutant p.(Val261Glyfs*135) (Mut1) is overexpressed (Figure 3) (Mattioli et al., 2020). The NOVA2 mutant protein p.(Leu252Phefs*144) behaves similarly to Mut1 as it fails to regulate these splicing events. However, the overexpression of the NOVA2 p.(Lys444Glnfs*82) protein, carrying the more distal frameshift, leads to an intermediate phenotype between WT and Mut1 NOVA2 proteins. This finding suggests that the p.(Lys444Glnfs*82) variant might have a milder effect than the other frameshift variants located just after the KH2 domain.

### 3.3 *NOVA2* variants cause a heterogeneous NDD with variable clinical severity

**Figure 4:**
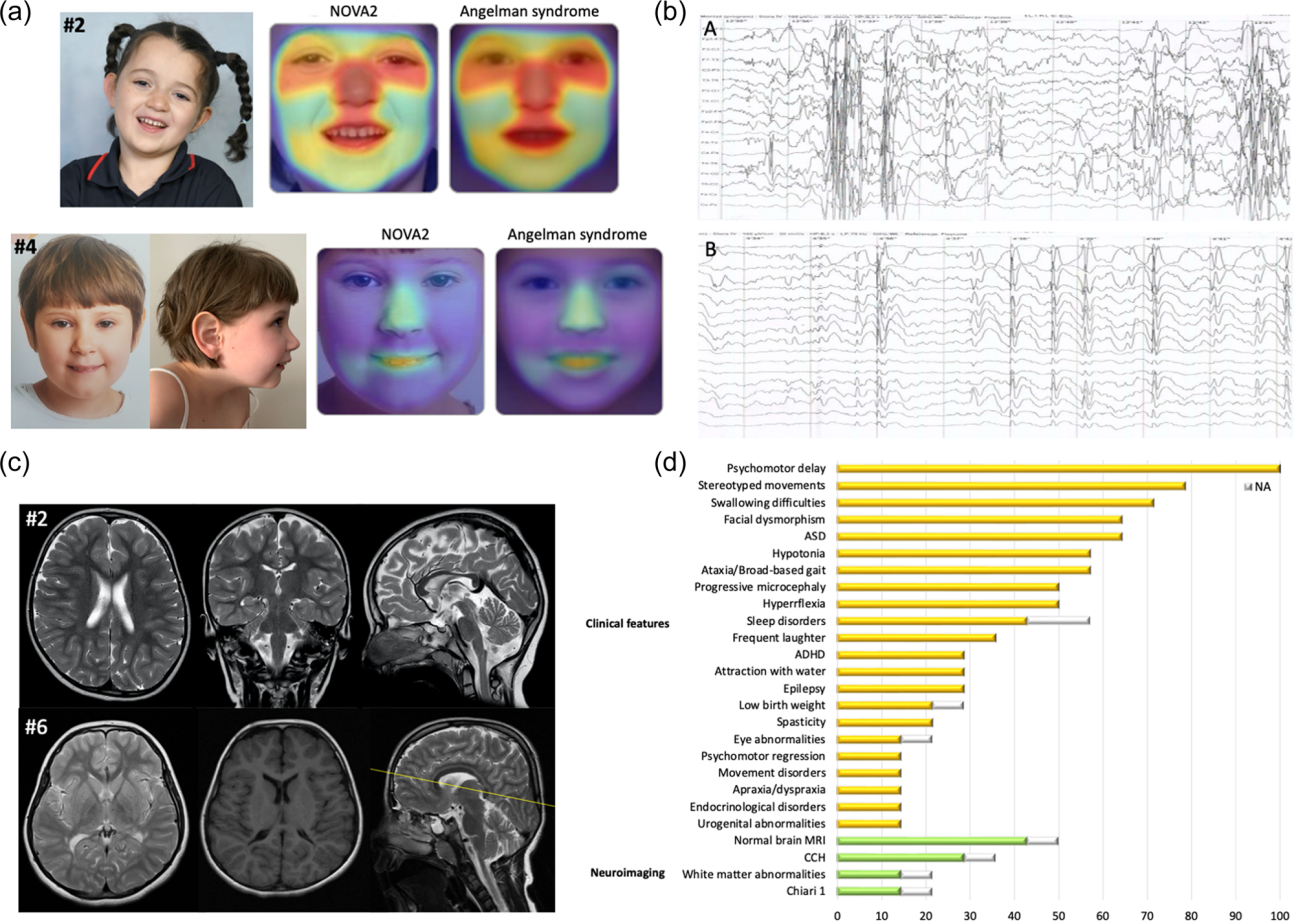
Electroclinical and neuroimaging features of patients harboring de novo truncating *NOVA2* variants. (a) Clinical photographs of patients #2 and #4. Patient #2 shows hypertelorism, intermittent esotropia, sunken nasal bridge, thin lips, and simplified protruding ears. Patient #4 shows slightly upslanting palpebral fissures, deep philtrum, large ears, and retrognathia with prominent chin. For each subject, a heat‐map comparison between the patient's frontal photograph and a composite picture from subjects with Angelman syndrome (Face2Gene, https://www.face2gene.com) is reported. (b) Electroencephalographic findings in patient #4. Ictal (A) and interictal (B) EEG showing generalized spike‐and‐wave activity and nonspecific electrical abnormalities, respectively. (c) Brain MRI (T2‐weighted sequences) of patient #2 at 5.7 years shows thinning of the posterior section of the body of the corpus callosum and prominent CSF spaces overlying both frontal lobes. Brain MRI (T1‐ and T2‐weighted sequences) of patient #6 at 11 years shows bilateral peritrigonal periventricular white matter volume loss with increased signal intensity and extension along the right corona radiata, associated with thinning of the posterior portion of the body of the corpus callosum. (d) Percentage distribution of recurrent clinical manifestations and neuroimaging findings in all reported *NOVA2* patients. Yellow and green bars indicate the percentage of patients with the corresponding clinical or neuroimaging feature, respectively. Grey bars indicate the percentage of patients in whom data were not available. ADHD, attention deficit‐hyperactivity disorder; ASD, autism spectrum disorder; EEG, electroencephalogram; CCH, corpus callosum hypoplasia; NA, not applicable.

The common neurodevelopmental phenotype observed in our cohort consisted of global psychomotor delay, consistently leading to moderate to severe intellectual disability, behavioral abnormalities, sleep disorders, and associated neurological features (Table 1). Neonatal course was unremarkable in most cases, but feeding problems due to swallowing difficulties were common (#1, #2, #6, #7, and #8). However, failure to thrive only occurred in one case (#6). In general, birth parameters were within normal ranges. Progressive microcephaly was instead observed in subjects #2 and #6‐8. Dysmorphic facial features were observed in #2, #3, #4, #6, and #7 (Figure 4a). A global developmental delay was noticed in the first year of life in all cases. Patient #7 was never able to walk, while the other subjects could walk, either assisted, or unassisted. Speech impairment was particularly significant in all cases, ranging from nonverbal (#2, #7, and #8) to a few words. Bowel and urinary incontinence were also observed in five cases (#1, #3, #4, #7, and #8). Behavioral abnormalities included autism spectrum disorder (ASD) (#2‐5, #7, and #8), attention deficit‐hyperactivity disorder (ADHD) (#5‐8), frequent laughter (#3, #4, and #8), and attraction with water (#2‐4). Most subjects showed variable stereotyped movements of the hands (#1, #2, #4, #5, and #7), especially in association with a state of arousal (Supporting Information: Video 1). These included hand flapping, wringing, and clapping. Additionally, body rocking and head banging were observed in patient #8. Sleep disturbances were also common, ranging from frequent awakenings (#2, #3, #7, and #8) to parasomnias (Supporting Information: Table 2).

**Table 1:** Summary of genetic, clinical, and neuroimaging features in subjects harboring de novo truncating *NOVA2* variants

Subject ID	#1	#2	#3	#4	#5	#6	#7	#8	Mattioli et al. (2020)^a^	Total
Age, sex	15.5 y, F	7 y, F	7 y, M	10 y, F	13 y, M	15 y, F	7 y, M	7 y, M	Mean 6.5 y, 6 cases (M/F = 1)	
Country of origin	Italy	Australia	Netherlands	Poland	Italy	Pakistan	German	German	France, USA	
*NOVA2*variant	c.787del (p.Ala263Profs*133)	c.754_756delCTGinsTT	c.1329dup	c.826del	c.523del	c.256C>T	c.755_764del	c.755_764del	c.701_720dup (p.Ala241Profs*162);	
(NM_002516.4)		(p.Leu252Phefs*144)	(p.Lys444Glnfs*82^b^)	(p.Leu276Cysfs*120)	(p.Leu175Cysfs*6)^b^	(p.Gln86*)^b^	(p.Leu252Profs*141)	(p.Leu252Profs*141)	c.709_748del (p.Val237Profs*146)	
Birth parameters	‐	‐	‐	‐	‐	‐	NA	NA	+ (3), NA (1)	3/14 (21.4%)
Low birth weight	‐	‐	NA	‐	‐	‐	NA	NA	NA (1)	0/14 (0%)
Congenital microcephaly										
Feeding difficulties										
Sucking/swallowing difficulties	+	+	‐	‐	‐	+	+	+	+ (5)	10/14 (71.4%)
GE reflux	‐	‐	‐	‐	+	‐	+	+	NA	3/14 (21.4%)
Failure to thrive	‐	‐	‐	‐	‐	+	‐	‐	+ (3)	4/14 (28.6%)
Psychomotor delay										
Motor delay	+	+	+	+	+	+	+	+	+ (6)	14/14 (100%)
Speech delay	+	+	+	+	+	+	+	+	+ (6)	14/14 (100%)
Bowel/urinary incontinence	+	‐	+	+	‐	‐	+	+	NA	5/14 (35.7%)
Intellectual disability (degree)	+ (severe)	+ (NA)	+ (severe)	+ (moderate)	+ (moderate)	+ (moderate)	+ (severe)	+ (severe)	+ (6)	14/14 (100%)
Psychomotor regression	‐	‐	‐	+	‐	+	‐	‐	‐	2/14 (14.3%)
Progressive microcephaly	‐	+	‐	‐	‐	+	+	+	+ (1), NA (1)	5/14 (35.7%)
Abnormal behavior										
ASD	‐	+	+	+	+	‐	+	+	+ (3)	9/14 (64.3%)
ADHD	‐	‐	‐	‐	+	+	+	+	‐	4/14 (28.6%)
Frequent laughter	‐	‐	+	+	‐	‐	‐	+	+ (2)	5/14 (35.7%)
Attraction with water	‐	+	+	+	‐	‐	‐	‐	+ (1)	4/14 (28.6%)
Stereotyped movements	+	+	‐	+	+	‐	+	+	+ (5)	11/14 (78.6%)
Neurological features										
Hypotonia	‐	‐	+	+	‐	+	+	+	+ (3), NA (2)	8/14 (57.1%)
Ataxia/broad‐based gait	+	+	‐	+	‐	+	+	NA	+ (3)	8/14 (57.1%)
Spasticity	‐	+	‐	‐	‐	+	‐	‐	+ (1)	3/14 (21.4%)
Hyperreflexia	+	+	‐	+	+	+	‐	‐	+ (2)	7/14 (50%)
Movement disorders	‐	‐	‐	+	‐	+	‐	‐	‐	2/14 (14.3%)
Apraxia/dyspraxia	‐	‐	‐	+	+	‐	‐	‐	‐	2/14 (14.3%)
Sleep disorders	+	+	+	‐	‐	‐	+	+	+ (1)	6/14 (42.8%)
Epilepsy									+ (2)	
Seizure onset	‐	‐	‐	4.5 y	‐	2 y	‐	‐	2.5‐9 y	
Seizure type	‐	‐	‐	MAS	‐	MS	‐	‐	MAS, SS (1); MS (1)	
EEG	Normal	Normal	NA	Generalized SWA	Normal	Normal	NA	NA	NA	4/14 (28.6%)
Response to AEDs	‐	‐	‐	Partial	‐	Partial	‐	‐	NA	
Seizure‐free	‐	‐	‐	No	‐	No	‐	‐	NA	
Syndromic features										
Facial dysmorphism	‐	+	+	+	‐	+	+	‐	+(4), NA (1)	9/14 (64.3%)
Eye abnormalities	‐	+	‐	‐	‐	‐	‐	‐	+ (1)	2/14 (14.3%)
GU abnormalities	‐	‐	+	+	‐	‐	‐	‐	‐	2/14 (14.3%)
Endocrine disorders	+	‐	‐	+	‐	‐	‐	‐	‐	2/14 (14.3%)
Brain MRI										
CCH	‐	+	NA	‐	‐	+	‐	‐	+ (2)	4/14 (28.6%)
Cortical atrophy	‐	‐	NA	‐	‐	‐	‐	‐	+ (1)	1/14 (7.1%)
WM abnormalities	‐	‐	NA	‐	‐	+	‐	‐	+ (1)	2/14 (14.3%)
Cerebellar abnormalities	‐	‐	NA	‐	‐	‐	‐	‐	+ (1)	1/14 (7.1%)
Chiari 1	‐	‐	NA	‐	+	‐	‐	‐	+ (1)	2/14 (14.3%)
Other	‐	+, prominent CSF spaces	NA	‐	‐	‐	‐	‐	Pineal cyst (1)	2/14 (14.3%)

Abbreviations: ADHD, attention deficit‐hyperactivity disorder; AEDs, antiepileptic drugs; ASD, autism spectrum disorder; CCH, corpus callosum hypoplasia; CSF, cerebrospinal fluid; EEG, electroencephalogram; GE, gastroesophageal; GU, genitourinary; MAS, myoclonic‐atonic/astatic seizures; MS, myoclonic seizures; NA, not applicable; SS, staring spells; SWA, spike‐and‐wave activity; WM, white matter.

^a^ PMID: 32197073.

^b^ Variants located outside the suggested “clustering region.”

In addition to the severe neurodevelopmental phenotype, psychomotor regression was observed in subjects #4 and #6. In particular, patient #4 experienced a loss of motor and verbal skills with increased epileptic activity, whereas no correlation with seizures could be observed in case #6. Interestingly, they also suffered from movement disorders, consisting of tremors (#4 and #6) and choreoathetosis (#4). Epileptic manifestations included refractory myoclonic or myoclonic‐atonic seizures with or without postictal state. Seizures were occasionally precipitated by sound stimuli, with an exaggerated startle response (startle seizures) (Supporting Information: Videos 1‐3), and associated with generalized spike‐and‐wave activity in subject #4 (Figure 4b), who showed a partial response to antiseizure medications. Muscle tone abnormalities consisted of hypotonia (#3, #4, #6‐8), spasticity (#2 and #6), or paratonia (#1). Five out of six subjects showed hyperreflexia (#1, #2, and #4‐6). Lack of coordination with unsteady gait and, in more severe cases, true ataxia was present (Supporting Information: Video 4). Two patients also presented with motor dyspraxia (#4 and #5).

Associated syndromic features included urogenital manifestations (intra‐abdominal testis in #3 and glomerulonephritis in #4), and endocrinological abnormalities (precocious puberty in #1 and hypothyroidism in #4). Brain magnetic resonance imaging (MRI) was normal in four cases (#1, #4, #7, and #8) while showed variable abnormalities in the remaining subjects, such as corpus callosum hypoplasia (CCH) (#2 and #6), bilateral peritrigonal periventricular white matter volume loss (#6), Chiari I malformation (#5), and prominent frontal subarachnoid spaces (#1) (Figure 4c).

## 4 DISCUSSION

The disruption of splicing regulation is involved in complex multifactorial diseases such as amyotrophic lateral sclerosis (ALS), mendelian disorders such as myotonic dystrophy (Lee & Rio, 2015; López‐Martínez et al., 2020), and specific NDDs such as *PUF60*‐ and *PQBP1*‐related disorders (OMIM #615583 and #309500, respectively) (El Chehadeh et al., 2016; Kalscheuer et al., 2003; La Cognata et al., 2020). Variants affecting AS have been associated with an increased risk for psychiatric features and are specifically involved in ASD, bipolar disorder, and schizophrenia (Cai et al. 2021; Parikshak et al., 2016; Paterson et al., 2017; Reble et al., 2018; Stamova et al., 2013; Weyn‐Vanhentenryck et al., 2018). In particular, the abnormal function of the tissue‐specific splicing regulator RNA‐binding protein FOX1 (RBFOX1, OMIM *605104), also known as Ataxin‐2‐Binding Protein 1 (A2BP1), plays a role in the modulation of developing cerebral cortex architecture and ASD pathophysiology (Hamada et al., 2015; Lee et al., 2016). Compelling evidence also implicate AS defects in the pathogenesis of neurodegenerative conditions, including Alzheimer's disease, Huntington's disease, Parkinson disease, and spinocerebellar ataxias (Apicco et al., 2019; Li et al., 2021).

NOVA2 is a crucial RBP for the AS regulation of several genes encoding proteins involved in neuronal differentiation and migration during brain development (Saito et al., 2019). A model in which a balance of transcript levels is maintained in the brain through a dynamically regulated *NOVA*‐dependent AS‐coupled NMD and direct interaction with 3' untranslated region binding elements has been recently suggested (Eom et al., 2013). This dynamism is well exemplified by the differential AS regulation of *ITPR1* (OMIM *147265), encoding the inositol 1,4,5‐triphosphate receptor type 1, in selected neuronal populations within different brain regions (Saito et al., 2019). NOVA2 not only operates as a *trans*‐acting AS factor to determine exon definition but also acts as a *cis*‐acting element regulating cell‐type specific retention of introns, which titrate the binding of other *trans*‐acting splicing factors (Saito et al., 2019). It is directly responsible for the AS regulation of several genes associated with NDDs (e.g., *AP1S2*) and other neurological conditions (e.g., *DAB1*) (Mattioli et al., 2020). Additionally, NOVA2 recently emerged as the first RBP to promote neural circular RNA (circRNA) biogenesis in the developing brain (Knupp et al., 2021; Patop et al., 2019; Tang et al., 2021). These molecules regulate gene transcription through microRNAs (miRNAs) repression and interaction with RBPs, modulating neuronal progenitor status maintenance, gene expression, synaptic transmission, and microglia activation (Gokool et al., 2020; Meng et al., 2019; Patop et al., 2019). Abnormal circRNA epigenetic modifications (especially 6‐methyladenosine, m6A) affect RNA stability and result in neuronal disorders (Meng et al., 2019; Zhang et al., 2019).

De novo truncating variants in *NOVA2* cause a severe NDD characterized by global psychomotor delay, behavioral disorders, stereotyped movements, poor motor coordination, feeding difficulties, and associated neurological features (Figure 4d). Dysmorphic features are common and include hypertelorism, deep‐set eyes with epicanthal folds and long eyelashes, large ears, pointed chin, and Angelman‐like features, such as sunken nasal bridge and long philtrum (Figure 4a). However, a specific facial gestalt does not appear to emerge. In addition to previously reported clinical features, variable novel manifestations were observed in our cohort, mainly consisting of ADHD, dyspraxia/apraxia, urogenital (intra‐abdominal testis, glomerulonephritis), and endocrinological (precocious puberty, hypothyroidism) abnormalities. Remarkably, we also observed an evident psychomotor regression in two cases (#4 and #6), correlating with a smallpox‐related increase in the epileptic activity in subject #4. These individuals also suffered from movement disorders consisting of tremors with or without choeroathetosis. Another previously unreported finding is the progressive microcephaly observed in four patients (#2 and #6‐8). Taken together, the phenotype extension suggests that this condition may have a heterogeneous and occasionally severe clinical course.

Although seizures have been previously reported in the two subjects harboring the p.(Val261Glyfs*135) and p.(Val261Trpfs*135) variants, the electroencephalographic findings and seizure response to medical treatment remained elusive (Mattioli et al., 2020). We observed myoclonic or myoclonic‐atonic seizures with onset at 2–4.5 years (Supporting Information: Table 2) and generalized spike‐and‐wave activity at the electroencephalogram in #4. Clonazepam alone was administered in subject #4, whereas patient #6 received a combination of topiramate, clobazam, and valproate. In both cases, only a partial clinical response consisting of decreased seizure frequency could be observed. Interestingly, seizures were occasionally precipitated by sound stimuli in subject #4, who also showed hyperekplexia, suggesting a differential diagnosis with reflex seizures (Striano et al., 2012). Cortical hyperexcitability and epilepsy have been observed in the heterozygous knock‐out mouse models, supporting an underlying pathogenic link between *NOVA2* function and epilepsy (Eom et al., 2013). More specifically, NOVA‐mediated regulation of NMD splicing controls the levels of many synaptic proteins (e.g., DLG3, PSD95) and ion channels/transporters (e.g., SCN9A, SLC4A10, and SLC4A3), whose abnormal stoichiometry can lead to electrical imbalance and epileptogenesis (Eom et al., 2013). Of note, NOVA2 was found to interact with a *cis*‐acting polymorphism in *SCN1A* (rs3812718) and modulate the proportions of drug‐responsive alternative transcripts (Heinzen et al., 2007).

Overall, brain MRI is normal in ~40% of *NOVA2* patients (6/14 subjects in total), whereas variable abnormalities are present in the remaining individuals (Table 1). Although some alterations could be observed in more than one subject (i.e., CCH, white matter volume loss, and Chiari I malformation), a common neuroimaging pattern cannot be delineated. More in general, the observed neuroimaging abnormalities might be the consequence of abnormal regulation of transcripts encoding crucial proteins for axonal growth and pathfinding resulting from NOVA2 deficiency (Saito et al., 2016). For example, several genes associated with CCH syndromes are target of NOVA2‐mediated AS regulation (e.g., *CASK* and *DCC*) and CCH is present in 33% of patients (Saito et al., 2016). However, this feature is nonspecific and the observation of a normal brain MRI in a not insignificant number of patients remains challenging to explain. Furthermore, while the progressive motor discoordination observed in the conditional mouse model with specifical *Nova2* inactivation is recapitulated in *NOVA2* patients, there is no comparable cerebellar atrophy (Saito et al., 2019). The report of an additional cohort will help further delineate the spectrum of *NOVA2*‐related brain abnormalities.

Among the variants identified in our cohort, four (p.(Leu252Profs*141), p.(Leu252Phefs*144), p.(Ala263Profs*133), and p.(Leu276Cysfs*120)) are located in proximity of the terminal of KH2 domain, in line with the previous report by Mattioli et al. (2020). The p.(Leu175Cysfs*6) variant is the first variant falling within the KH2 domain to be reported. Similarly, we identified the first *NOVA2* variants localizing to the KH1, p.(Gln86*), and KH3 domain, p.(Lys444Glnfs*82). Of note, the c.256C>T p.(Gln86*) variant is the first nonframeshift *NOVA2* variant to be described and the first variant localized in Exon 3, whereas all other reported variants fall in Exon 4 (Figure 1). The four variants localized to the “cluster region” (p.(Leu252Profs*141), p.(Leu252Phefs*144), p.(Ala263Profs*133), and p.(Leu276Cysfs*120)) (Figure 1) lead to the same alternative frame of previously reported changes (Mattioli et al., 2020). The remaining variants are instead predicted to cause different functional consequences. While the p.(Leu175Cysfs*6) likely disrupts the KH2 domain and the distal p.(Lys444Glnfs*82) results in a more preserved alternative C‐terminal of the protein, the early p.(Gln86*) variant very likely results in NMD. This partially questions the previously assumed parallel between *NOVA2*‐related disorder and Robinow syndrome as conditions caused by specific distal frameshift variants (Mattioli et al., 2020; Supek et al., 2020). However, although puzzling, the localization pattern of all known *NOVA2* variants and the observation that most of them lead to the same alternative frame still suggests the existence of a possible mutational hotspot region (Figure 1).

Although a possible gain‐of‐function effect cannot be excluded, *NOVA2* variants are predicted to act through a partial loss of function (hypomorphic) mechanism (Mattioli et al., 2020). However, the functional consequences of proximal p.(Gln86*) and distal p.(Lys444Glnfs*82) variants might fall at the opposite ends of the pathophysiological spectrum. The addition of the C‐terminal part produced by the recurrent frameshift variants next to the KH2 domain allows a residual function of the protein (Mattioli et al., 2020). An early truncating variant is instead likely to result in a severe impairment of protein function. This is exemplified by the much stronger loss of AS regulation produced by the overexpression of the truncated p.Tyr231* NOVA2 protein variant in HeLa cells as compared with Mut1 (Mattioli et al., 2020). In line with this observation, the p.(Gln86*) is expected to result in a complete loss of NOVA2 function and potentially lead to more severe consequences than other variants. The p.(Lys444Glnfs*82) variant might instead lead to more subtle functional effects, sparing the KH3 domain and allowing the production of a more functionally active truncated protein. This is also supported by the milder impact of this variant on AS events, resulting in an intermediate phenotype between WT and Mut1 p.(Val261Glyfs*135). Indeed, the KH3 domain is crucial for the binding to the UCAY sequence in the pre‐mRNA, which cannot be duplicated by KH1 and KH2 domains (Jensen et al., 2000; Lewis et al., 2000).

The spectrum of *NOVA2* variants might be wider than previously expected and their localization might influence the residual protein function. Although it is tempting to speculate that the neurological phenotype (epilepsy and psychomotor regression) associated with the proximal p.(Gln86*) variant is more severe than that related to the distal p.(Lys444Glnfs*82), similar features were also observed in the subject harboring the p.(Leu276Cysfs*120) variant (Supporting Information: Table 2). Additionally, the clinical manifestations of patients harboring variants localizing outside of the “clustering region” appear to be quite overlapping with the other subjects except for ADHD, which was never reported in patients with *NOVA2* variants before. To further complicate the picture, a possible NMD escape cannot be completely excluded for the proximal p.(Gln86*) variant, making it premature to draw any conclusions (Inácio et al., 2004; Pereira et al., 2015; Dyle et al., 2020). In light of these observations, the report of other cohorts expanding the molecular spectrum of *NOVA2*‐related disorder will likely play a crucial role in the delineation of potential genotype‐phenotype correlations.

## 5 CONCLUSION

De novo truncating *NOVA2* variants lead to a severe and heterogeneous neurodevelopmental condition with behavioral disturbances, epilepsy, neurological features, and variable brain MRI abnormalities. Our findings confirm that pathogenic *NOVA2* variants negatively affect AS events, likely leading to impaired neuronal development, axon guidance, and synaptic plasticity and function. The milder functional impairment observed for the distal p.(Lys444Glnfs*82) variant suggests that variant localization might influence the residual protein function, possibly determining a wider than expected molecular and phenotypic spectrum. However, this intermediate effect detected in vitro does not necessarily predict an intermediate effect in vivo in a physiological context. Moreover, the limited number of reported subjects and the complex mechanisms involved in NMD escape make it difficult to draw conclusions on the pathophysiological link between specific variants and phenotypic manifestations. Further studies will hopefully help clarify these intriguing aspects, possibly laying the foundation for more robust genotype‐phenotype correlations in *NOVA2* patients.

## WEB RESOURCES

DECIPHER: https://decipher.sanger.ac.uk

Ensembl; https://www.ensembl.org/index.html

Gene Cards: http://www.genecards.org

Gene Matcher: http://www.genematcher.org

Genome Aggregation Database (GnomAD): http://gnomad.broadinstitute.org

Leiden Open Variation Database (LOVD): https://www.lovd.nl

Mutation Taster: http://www.mutationtaster.org

Online Mendelian Inheritance in Man: http://www.ncbi.nlm.nih.gov/Omim

Proteomics DB: https://www.proteomicsdb.org

PubMed: http://www.ncbi.nlm.nih.gov/pubmed

Human Genome Variation Society: https://varnomen.hgvs.org

RefSeq: https://www.ncbi.nlm.nih.gov/refseq

SIFT: https://sift.bii.a-star.edu.sg

The 1000 Genomes Browser: http://browser.1000genomes.org/index.html

UCSC Human Genome Database: http://www.genome.ucsc.edu

UniProt: https://www.uniprot.org Var some: https://varsome.com

Varsome: https://varsome.com

## CONFLICT OF INTEREST

The authors declare no conflict of interest.

## Supporting information

Supporting information.

Supporting information.

Supporting information.

Supporting information.

Supporting information.

Supporting information.

Supporting information.

## Data Availability

All variants were submitted to the Leiden Open Variation Database (LOVD, https://www.lovd.nl) with the following accession numbers: #0000797459, #0000797460, #0000797461, #0000797462, #0000797463, #0000797464, and #0000841619.
