# Supplementary material for: De novo truncating NOVA2 variants affect alternative splicing and lead to heterogeneous neurodevelopmental phenotypes
Source: Hum Mutat. 2022 Jun 8;43(9):1299–313. doi: 10.1002/humu.24414 (PMC9543825; doi:10.1002/humu.24414)
Supplement: Supplementary file 1 — Supporting information. [file HUMU-43-1299-s007.docx]

**Supplementary Material**

**1. Supplementary Methods**

**2. Supplementary Videos**

**3. Supplementary Tables**

**4. Supplementary References**

**1. Supplementary Methods**

**Genetic testing and data analysis**

After standard DNA extraction, trio exome sequencing (ES) was performed in all cases. Patient #1 was sequenced within the Autism Sequencing Consortium (ASC) collaborative network. The quality of the sequence reads was assessed through QC statistics with FastQC (<http://www.bioinformatics.bbsrc.ac.uk/projects/fastqc>). Reads alignment to the reference human genome (hg19, UCSC assembly, February 2009) was performed through BWA with default parameters (Li and Durbin, 2009). HaplotypeCaller algorithm within the GATK package was employed for quality score recalibration, indel realignment, and variant calling (DePristo et al., 2011; McKenna et al., 2010). Variants were then annotated with ANNOVAR (Wang et al., 2010). After being filtered out for minor allele frequency (MAF) ≤ 0.001 in GnomAD (<https://gnomad.broadinstitute.org>) and in our in-house database of 3,000 exomes, the predicted impact of the candidate variants on protein structure and function was evaluated through *in silico* prediction tools. The most interesting candidate variants were confirmed through Sanger sequencing, which was performed according to standard procedures (Tarailo-Graovac et al., 2016). Sanger sequencing was also performed for parental segregation analysis confirmation. chromosomal microarray analysis (CMA) was performed in #1, #5, #7, and #8 as previously described (Redon et al., 2009). All variants are reported according to the NCBI Reference Sequence NM_002516.4, GenBank NC_000019.10 (<https://www.ncbi.nlm.nih.gov/nuccore/NM_002516.4>).

**Facial dysmorphism analysis and heat-map comparison**

The frontal images of individuals #2 and #4 were analyzed using Face2Gene technology (FDNA Inc., Boston, MA, USA; <https://www.face2gene.com>). No clinical photograph was available for other patients. For each of the analyzed subjects, the first syndrome to be suggested based on the facial dysmorphism was Angelman syndrome. In order to highlight the most relevant overlapping dysmorphic features between these two conditions, we then performed a heat-map comparison between the frontal photographs of the studied patients and a composite picture obtained from subjects with Angelman syndrome. For each individual, the obtained heatmap was included in Figure 1.

**2. Supplementary Videos**

**Supplementary video 1.** Subject #4 at the age of 10 years, in the hospital, shows hand waving and wringing when excited. The patient experiences an exaggerated reaction to the sound stimulus, followed by sudden stiffening and fall, suggestive of tonic startle seizure (abnormal EEG).

**Supplementary videos 2 and 3.** Subject #4 at the age of 10 years, at home, shows an abnormal startle response to sound stimuli, characterized by sudden stiffening and babbling.

**Supplementary video 4.** Subject #4 at the age of 10 years, walking outside, shows broad-based gait and easy fatigability. She walks with forward tilting and flexed upper arms to compensate lack of balance.

**3. Supplementary Tables**

**Supplementary Table 1.** Detailed phenotypic delineation of new affected individuals.

**Supplementary Table 2.** Extensive *in silico* analysis of novel *NOVA2* variants.

**4. Supplementary References**

DePristo, M. A., Banks, E., Poplin, R., Garimella, K. V., Maguire, J. R., Hartl, C., Philippakis, A.

A., del Angel, G., Rivas, M. A., Hanna, M., McKenna, A., Fennell, T. J., Kernytsky, A. M., Sivachenko, A. Y., Cibulskis, K., Gabriel, S. B., Altshuler, D., Daly, M. J. (2011). A framework for variation discovery and genotyping using next-generation DNA sequencing data. *Nat Genet, 43*, 491-498. <https://doi.org/10.1038/ng.806>

Li, H., & Durbin, R. (2009). Fast and accurate short read alignment with Burrows-Wheeler

transform. *Bioinformatics, 25*, 1754-1760. <https://doi.org/10.1093/bioinformatics/btp324>

McKenna, A., Hanna, M., Banks, E., Sivachenko, A., Cibulskis, K., Kernytsky, A., Garimella, K.,

Altshuler, D., Gabriel, S., Daly, M., DePristo, M.A. (2010). The Genome Analysis Toolkit: a MapReduce framework for analyzing next-generation DNA sequencing data. *Genome Res, 20*, 1297-1303. <https://doi.org/10.1101/gr.107524.110>

Redon, R., & Carter, N.P. Comparative genomic hybridization: microarray design and data

interpretation. (2009). *Methods Mol Biol, 529*, 37-49. <https://doi.org/10.1007/978-1-59745-538-1_3>

Tarailo-Graovac, M., Shyr, C., Ross, C. J., Horvath, G. A., Salvarinova, R., Ye, X. C., Zhang, L.

H., Bhavsar, A. P., Lee, J. J., Drögemöller, B. I., Abdelsayed, M., Alfadhel, M., Armstrong, L., Baumgartner, M. R., Burda, P., Connolly, M. B., Cameron, J., Demos, M., Dewan, T., … van Karnebeek, C. D. (2016). Exome Sequencing and the Management of Neurometabolic Disorders. *N Engl J Med, 374*, 2246-2255. <https://doi.org/10.1056/NEJMoa1515792>

Wang, K., Li, M., Hakonarson, H. (2010). ANNOVAR: functional annotation of genetic variants

from high-throughput sequencing data. *Nucleic Acids Res, 38*, e164. <https://doi.org/10.1093/nar/gkq603>
